# Vpx rescues HIV-1 transduction of dendritic cells from the antiviral state established by type 1 interferon

**DOI:** 10.1186/1742-4690-8-49

**Published:** 2011-06-22

**Authors:** Thomas Pertel, Christian Reinhard, Jeremy Luban

**Affiliations:** 1 Department of Microbiology and Molecular Medicine, University of Geneva, Geneva, Switzerland

## Abstract

**Background:**

Vpx is a virion-associated protein encoded by SIV_SM_, a lentivirus endemic to the West African sooty mangabey (*Cercocebus atys*). HIV-2 and SIV_MAC_, zoonoses resulting from SIV_SM _transmission to humans or Asian rhesus macaques (*Macaca mulatta*), also encode Vpx. In myeloid cells, Vpx promotes reverse transcription and transduction by these viruses. This activity correlates with Vpx binding to DCAF1 (VPRBP) and association with the DDB1/RBX1/CUL4A E3 ubiquitin ligase complex. When delivered experimentally to myeloid cells using VSV G-pseudotyped virus-like particles (VLPs), Vpx promotes reverse transcription of retroviruses that do not normally encode Vpx.

**Results:**

Here we show that Vpx has the extraordinary ability to completely rescue HIV-1 transduction of human monocyte-derived dendritic cells (MDDCs) from the potent antiviral state established by prior treatment with exogenous type 1 interferon (IFN). The magnitude of rescue was up to 1,000-fold, depending on the blood donor, and was also observed after induction of endogenous IFN and IFN-stimulated genes (ISGs) by LPS, poly(I:C), or poly(dA:dT). The effect was relatively specific in that Vpx-associated suppression of soluble IFN-β production, of mRNA levels for ISGs, or of cell surface markers for MDDC differentiation, was not detected. Vpx did not rescue HIV-2 or SIV_MAC _transduction from the antiviral state, even in the presence of SIV_MAC _or HIV-2 VLPs bearing additional Vpx, or in the presence of HIV-1 VLPs bearing all accessory genes. In contrast to the effect of Vpx on transduction of untreated MDDCs, HIV-1 rescue from the antiviral state was not dependent upon Vpx interaction with DCAF1 or on the presence of DCAF1 within the MDDC target cells. Additionally, although Vpx increased the level of HIV-1 reverse transcripts in MDDCs to the same extent whether or not MDDCs were treated with IFN or LPS, Vpx rescued a block specific to the antiviral state that occurred after HIV-1 cDNA penetrated the nucleus.

**Conclusion:**

Vpx provides a tool for the characterization of a potent, new HIV-1 restriction activity, which acts in the nucleus of type 1 IFN-treated dendritic cells.

## Background

In addition to the *gag*, *pol*, and *env *genes common to all retroviruses, lentiviruses including HIV-1 bear specialized genes such as *vpr *that contribute to viral replication and pathogenesis [1]. Simian immunodeficiency viruses isolated from West African sooty mangabeys (SIV_SM_) possess *vpr *as well as a highly homologous gene called *vpx*. The latter may have been generated by *vpr *gene duplication [2] or by recombination with an SIV that possessed a highly divergent *vpx *[3]. HIV-2 and SIV_MAC_, zoonoses derived from SIV_SM_, also possess both of these genes.

Neither *vpr *nor *vpx *is essential for virus replication in tissue culture, but both contribute to virus replication and disease progression in animal models [4,5]. The effect of these genes *in vivo *is possibly linked to their ability to enhance virus replication in dendritic cells and macrophages in tissue culture [6-15]. Myeloid cells are believed to be critical targets for lentiviruses *in vivo*, partly because they are capable of productive infection, but also because they facilitate virus transmission to CD4^+ ^T-cells [16-18].

Via interaction with short peptide signals in the carboxy-terminus of the Gag polyprotein, the Vpr and Vpx proteins are incorporated into nascent virions as the particles exit productively infected cells [19-22]. The presence of these proteins within virions suggests that they play a role in the early steps of lentivirus infection, prior to *de novo *protein synthesis directed by transcripts from the new provirus. Vpr and Vpx promote reverse transcription soon after the virions enter the target cell cytoplasm [10,13,14]. Other studies suggested that Vpr and Vpx are required later in the retrovirus life cycle to promote nuclear import of the preintegration complex [23-26], though the significance of the latter findings have been questioned [27,28].

Attempts to saturate a hypothetical HIV-1-specific restriction factor in monocyte-derived dendritic cells (MDDC) using HIV-1 VLPs have led to the fortuitous discovery that SIV_MAC _VLPs increase HIV-1 reverse transcription and infectivity in these cells, so long as the VLPs possess Vpx [10,11,29]. Similar stimulation of infectivity was observed with proteasome inhibitors, suggesting that Vpx promotes the degradation of an antiviral factor; CUL5-dependent degradation of the antiviral protein APOBEC3G by the lentiviral accessory protein Vif offered compelling precedent for such a model [30-32]. Indeed, heterokaryon experiments suggested that myeloid cells possess a dominant-acting, Vpx-sensitive inhibitor of lentiviral infection [12]. Via direct binding to DCAF1 (also known as VPRBP), both Vpr and Vpx associated with the DDB1/RBX1/CUL4A E3 ubiquitin ligase complex [12,13,15,33-37]. Vpx mutants that do not bind DCAF1 are unable to stimulate infectivity in myeloid cells [12,13,15].

Here, we report the results of experiments designed to determine the effect of Vpx on HIV-1 transduction of MDDCs in the face of the potent antiviral state pre-established by treatment with exogenous type 1 interferon (IFN) or with agonists of pattern recognition receptor (PRRs) that stimulate endogenous type 1 IFN production and the transcription of interferon stimulated genes (ISGs).

## Results

### SIV_MAC _VLPs rescue HIV-1 infection from type I IFN

The Vpx proteins of SIV_MAC _and HIV-2 promote transduction of myeloid cells by these viruses [6-15]. Though HIV-1 does not possess a gene encoding Vpx, the infectivity of HIV-1 in myeloid cells is also increased by SIV virus-like particles (VLPs) bearing Vpx [10,11,29]. Interest in potential links between retroviral restriction factors and innate immune signaling [38,39] directed us to explore the effect of Vpx on HIV-1 transduction of myeloid cells after an antiviral state had been established by administration of exogenous type 1 IFN.

**Figure 1:**
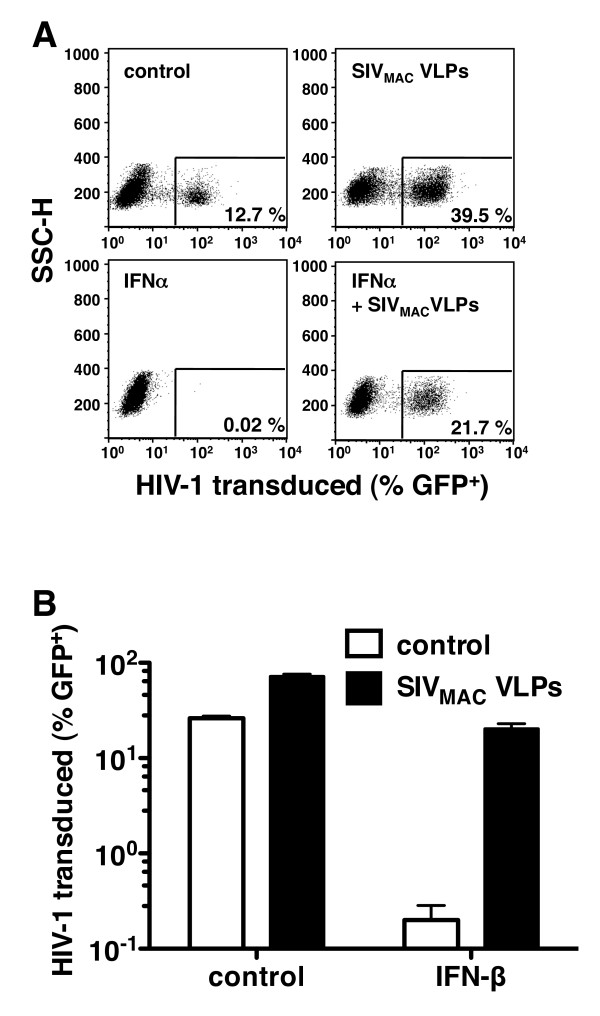
**SIV_MAC _virus-like particles (VLPs) rescue HIV-1 transduction of human monocyte-derived dendritic cells (MDDCs) from pretreatment with type I ifN**. MDDCs were incubated for 24 h with 10 ng⁄mL IFN-α (**A**) or 10 ng⁄mL IFN-β (**B**). The cells were then treated for 3 h with media or VSV-G-pseudotyped SIV_MAC-251 _VLPs, followed by challenge with a VSV-G-pseudotyped HIV-1_NL4-3 _GFP reporter virus. The percent GFP-positive cells was determined by flow cytometry 72 h after transduction. Error bars represent ± standard deviation (SD) (*n *= 3). In each case, one representative example of at least three independent experiments is shown.

Human monocyte-derived dendritic cells (MDDC) were generated by culture of CD14^+ ^peripheral blood cells in GM-CSF and IL-4 for 4 days, as previously described [39]. The status of differentiation and maturation was confirmed by observing the typical morphology and by assessing immunofluorescence for standard cell surface markers, including CD1A, CD209 (DC-SIGN), CD14, CD11C, HLA-DR, CD83, and CD86 (additional file 1, Figure S1A and data not shown). When immature MDDCs were challenged with three-part, HIV-1-GFP reporter virus, pseudotyped with vesicular stomatitis virus glycoprotein (VSV G), SIV_MAC _VLPs increased transduction efficiency 3- to 10-fold (Figure 1A, upper panels), depending upon the multiplicity of infection. Challenge of MDDC with HIV-1-GFP 24 h after treatment with exogenous IFN-α resulted in infection levels at or below the detection limit (Figure 1A, lower left panel). In the particular experiment shown in Figure 1, the magnitude inhibition of HIV-1 transduction by IFN-α was ≥ 600-fold. Addition of SIV_MAC _VLPs to the MDDCs 24 h after IFN-α treatment rescued HIV-1 transduction to levels at least as high as those in the absence of IFN-α (Figure 1A, lower right panel). Identical results were obtained when IFN-β was substituted for IFN-α (Figure 1B).

### SIV_MAC _VLPs rescue HIV-1 transduction of MDDC from LPS, poly(I:C), or poly(dA:dT)

Lipopolysaccharide (LPS), the synthetic double-stranded RNA poly(I:C), and the synthetic double-stranded DNA, poly(dA:dT), each activate *IFNB1 *transcription and establish a generalized antiviral state [39-42]. Treatment of MDDC with LPS, poly(I:C), or poly(dA:dT) indeed resulted in the production of soluble IFN-β (additional file 1, Figure S1B), the synthesis of intracellular MX1 and APOBEC3A proteins (additional file 1, Figure S1C), the transcriptional induction of *IFNB1 *and other inflammatory genes, including *MX1*, *CCL2*, *CCL8*, *CXCL10*, *IL6*, *ISG54 (IFIT2)*, *PTGS2*, and *TNF *(additional file 1, Figure S1D), as well as the upregulation of MDDC cell surface maturation markers, including CD86 and CD83 (additional file 1, Figure S1A).

**Figure 2:**
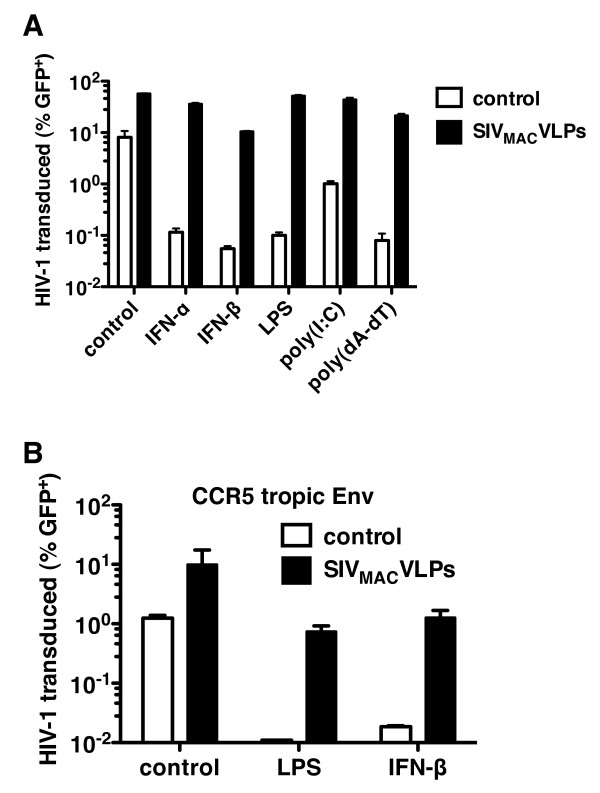
**SIV_MAC _VLPs rescue HIV-1 infectivity from pretreatment of MDDC with pattern recognition receptor (PRR) agonists**. (**A) **MDDCs were incubated for 24 h with recombinant type I interferon (10 ng⁄mL IFN-α, 10 ng⁄mL IFN-β), or PRR agonists as indicated: 100 ng⁄mL LPS, 25 μg⁄mL poly(I:C) with no lipid carrier, or 2 μg⁄mL poly(dA-dT). Then, cells were treated for 3 h with media or VSV-G-pseudotyped SIV_MAC-251 _VLPs, followed by challenge with a VSV G-pseudotyped HIV-1_NL4-3 _GFP reporter virus (A) or with a CCR5-tropic, HIV-1_NL4-3 _GFP reporter virus (B). The percent GFP-positive cells was determined by flow cytometry 72 h after addition of the reporter virus. Error bars represent ± SD (*n *= 3). In each case, one representative example of at least three independent experiments is shown.

Since LPS, poly(I:C), and poly(dA:dT) all elicited type 1 IFN in MDDCs, the ability of each to inhibit HIV-1 transduction was examined. MDDCs were treated for 24 h with either LPS, poly(I:C), or poly(dA:dT) and then challenged with VSV G-pseudotyped HIV-1-GFP reporter virus. Each of the treatments potently inhibited HIV-1-GFP transduction (Figure 2A). When SIV_MAC _VLPs were added to the culture 24 h after treatment with any of the PRR agonists, HIV-1-GFP two-part vector transduction was rescued completely (Figure 2A). Similar results were observed when HIV-1 entry was mediated by CCR5-tropic HIV-1 Env, indicating that the effect of Vpx was not peculiar to VSV G-pseudotyped HIV-1 (Figure 2B). The SIV_MAC _VLPs had no detectable effect on IFN-β secretion, MX1 or APOBEC3A protein production, cell-surface levels of MDDC maturation markers, or mRNA induction of *IFNB1 *and a panel of 8 ISGs (additional file 1, Figure S1). These findings indicate that the effect of the SIV_MAC _VLPs was relatively specific and that the VLPs did not globally reverse the antiviral state associated with type 1 IFN.

### Vpx is necessary and sufficient to protect HIV-1 from the type I IFN response

**Figure 3:**
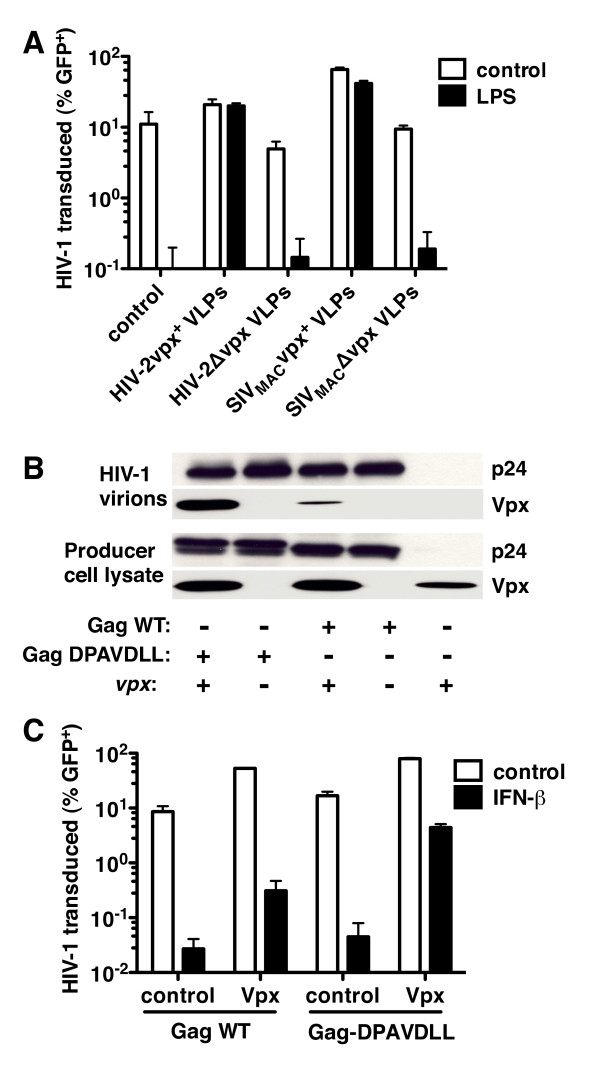
**Among VLP constituents, Vpx is necessary and sufficient to rescue HIV-1 from type I IFN**. (**A**) MDDCs were treated with LPS for 24 hrs, then treated for 3 hrs with media or the indicated VSV-G-pseudotyped HIV-2_ROD _or SIV_MAC-251 _VLPs, and finally challenged with a VSV-G-pseudotyped HIV-1_NL4-3 _GFP reporter virus. Infectivity was measured by flow cytometry. (**B**) As indicated, 293T cells were co-transfected with a codon optimized SIV_MAC251 _*vpx *expression plasmid and HIV-1 GFP reporter vectors bearing either wild-type Gag or Gag with an engineered Vpx binding motif (DPAVDLL). Proteins from the cell lysate and from virion preparations were separated by SDS-PAGE and then immunoblotted with anti-Vpx or anti-p24 antibodies. (**C**) MDDCs treated with IFN-β for 24 h and were then challenged with VSV-G-pseudotyped HIV-1 GFP reporter vectors with wild-type HIV-1 Gag or HIV-1 Gag bearing the engineered Vpx binding motif (DPAVDLL). Both HIV-1 reporter vectors were produced in the presence of empty pcDNA3.1 plasmid or pcDNA3.1 containing a codon-optimized SIV_MAC-251 _*vpx *cDNA. Data are representative of one of at least three independent experiments. Error bars represent ± SD (n = 3).

Vpx is essential for the boost in HIV-1 transduction of human MDDCs that is provided by SIV_MAC _VLPs [10,11,29]. To determine if Vpx is also required for the protective effect of VLPs in the context of the type 1 IFN-associated antiviral state, VLPs bearing Vpx were compared with VLPs lacking Vpx. Either SIV_MAC _VLPs or HIV-2 VLPs rescued a three-part HIV-1 vector from type 1 IFN or LPS treatment in human MDDC, but only when Vpx was present (Figure 3A). The same results were obtained if *vpx *was provided *in cis *or *in trans *with respect to the SIV structural proteins during assembly of the VLPs (additional file 2, Figure S2A), if Vpx was delivered by VLPs or whole SIV virus (additional file 2, Figure S2B), or if Vpx was encoded by HIV-2_ROD_, SIV_MAC251_, SIV_MAC239_, or SIV_SMM-PBJ _(data not shown). Vpr encoded by SIV_MAC_, HIV-2, SIV_AGM _or HIV-1 did not rescue HIV-1 from the antiviral state and, if anything, decreased the efficiency of rescue by Vpx (additional file 2, Figure S2B).

HIV-1 Gag p6 lacks the carboxy-terminal D-X-A-X-X-L-L peptide found in SIV_MAC _and HIV-2 Gag that confers optimal Vpx incorporation into virions [19-21,43,44]. Nonetheless, *vpx *expression *in trans *during HIV-1 virion production has been reported to result in some Vpx protein incorporation into HIV-1 virions with concomitant increase in the efficiency of MDM transduction by HIV-1 [12]. Vpx protein production directed by a codon-optimized *vpx *expression plasmid during HIV-1 virion production resulted in detectable Vpx incorporation into HIV-1 virions (Figure 3B) and partial rescue of HIV-1 three-part vector transduction in MDDCs that had been treated 24 hrs previously with IFN-β (Figure 3C). When the Vpx binding motif from the carboxy terminus of SIV_MAC _Gag (DPAVDLL) was engineered into HIV-1 Gag, Vpx packaging into HIV-1 virions was more efficient (Figure 3B) and rescue from IFN-β by Vpx was 10-fold more effective than it was with the parent construct (Figure 3C). These results indicate that, of the SIV_MAC _VLP components, Vpx is sufficient to rescue HIV-1 transduction from the type 1 IFN-associated antiviral state in MDDCs.

### Vpx does not rescue HIV-2 or SIV_MAC _from the antiviral state

**Figure 4:**
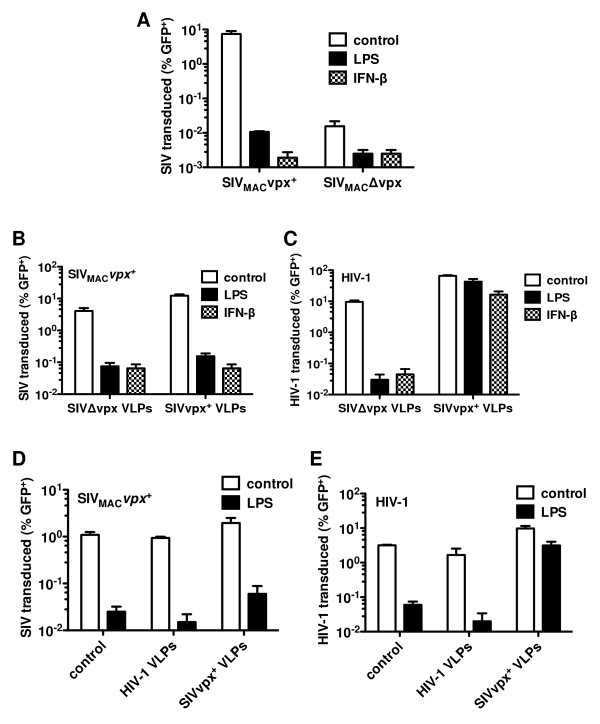
**Vpx rescues HIV-1, but not SIV_MAC _or HIV-2, from the type I IFN response in MDDC**. (**A**) MDDCs were treated with the indicated compounds for 24 h, and then challenged with VSV-G-pseudotyped, *vpx*^+ ^or Δ*vpx *SIV_MAC _GFP reporter virus. (**B, C**) MDDCs were treated with the indicated compounds for 24 h, then treated with either VSV-G-pseudotyped *vpx*^+ ^or Δ*vpx *SIV_MAC-251 _VLPs, and then challenged with either VSV-G-pseudotyped SIV_MAC-239 _(**B**) or HIV-1_NL4-3 _(**C**) GFP reporter viruses. (**D, E**) MDDCs were treated with LPS, then treated with either media or VSV-G pseudotyped HIV-1_NL4-3 _or SIV_MAC-239 _VLPs (containing all accessory genes) for 3 h, and then challenged with either VSV-G-pseudotyped SIV_MAC-239 _(**D**) or HIV-1_NL4-3 _(**E**) GFP reporter viruses. Data are representative of one of at least three independent experiments. Error bars represent ± SD (n = 3).

As previously described [6,7,11,45], disruption of the *vpx *open reading frame severely attenuated the transduction of MDDCs by three-part SIV_MAC _vector (Figure 4A), confirming the importance of *vpx *for MDDC-transduction in the absence of exogenous type 1 IFN or LPS. In contrast, when an antiviral state was established with exogenous IFN or LPS prior to virus challenge, *vpx *did not rescue transduction by SIV_MAC _or HIV-2, even when SIV_MAC _or HIV-2 VLPs provided additional Vpx *in trans *(Figure 4 and additional file 3, Figure S3); in parallel, the same SIV_MAC _VLPs rescued HIV-1 transduction from the antiviral state in a *vpx*-dependent fashion (Figure 4C). Additionally, HIV-1 VLPs bearing all HIV-1 accessory genes were unable to rescue either HIV-1 or SIV_MAC _from the antiviral state (Figure 4D and 4E). These experiments demonstrate that Vpx has the ability to rescue HIV-1, but not SIV_MAC _from the antiviral state.

### Rescue of HIV-1 from the antiviral state by Vpx is independent of DCAF1

**Figure 5:**
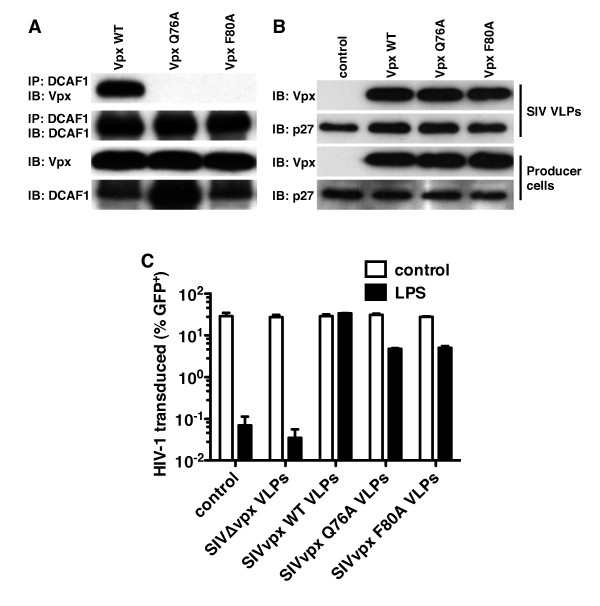
**SIV_MAC _Vpx association with DCAF1 (VPRBP) is dispensable for Vpx-mediated rescue of HIV-1 from the antiviral state**. (**A**) 293T cells were transfected with FLAG-tagged DCAF1 and either wild type SIV_MAC-251 _Vpx or SIV_MAC-251 _Vpx containing the indicated alanine-substitution mutations that disrupt associated with DCAF1. Immune complexes were isolated from clarified, 0.5% CHAPSO detergent lysates using anti-FLAG antibody conjugated to Protein G magnetic beads. Panels show immunoblots (IB) of the immunoprecipiated (IP) proteins (top panels) and immunoblots of the inputs (bottom panels). (**B**) Immunoblots of wild-type Vpx and the indicated mutants incorporated into SIV_MAC-251 _VLPs (top panels) and expression in the 293T producer cells (bottom panels). (**C**) MDDCs were treated with LPS, then treated with SIV_MAC-251 _VLPs containing wild-type Vpx or the indicated mutants, and challenged with an HIV-1_NL4-3 _GFP reporter virus. Data represent one of at least three independent experiments. Error bars represent ± SD (n = 3).

Vpx associates with the DDB1/RBX1/CUL4A E3 ubiquitin ligase complex via interaction with DCAF1 [12,13,15]. SIV_MAC _replication in macrophages is compromised by disruption of Vpx association with DCAF1 using *vpx *mutations Q76A or F80A, or by knockdown of DCAF1 or components of the DDB1/RBX1/CUL4A complex [12,13,15,35]. To address the role of DCAF1 and the associated E3 ubiquitin ligase complex in rescue of HIV-1 from the antiviral state in MDDCs, the Q76A and F80A *vpx *mutations were introduced into a codon-optimized SIV_MAC _*vpx *expression construct. Both mutant proteins expressed as well as wild type Vpx (Figure 5A) and were efficiently incorporated into SIV_MAC _VLPs (Figure 5B). As compared to the wild-type Vpx, the efficiency of HIV-1 rescue from the antiviral state in MDDCs by either mutant was reduced roughly 5-fold (Figure 5C). Nonetheless, both mutants retained the ability to rescue HIV-1 from the antiviral state 140-fold (Figure 5C), indicating that interaction with DCAF1 is not required for this activity.

The importance of DCAF1 for *vpx*-mediated rescue from the antiviral state was examined directly by transducing MDDCs with lentiviral vectors engineered to confer puromycin-resistance and to express RNA polymerase II-driven, microRNA-based short hairpin RNAs targeting either DCAF1 or a control RNA [39,46]. Freshly isolated CD14^+ ^monocytes were transduced in the presence of SIV_MAC _VLPs to increase the effective titer of the knockdown vectors. Cells were placed in GM-CSF and IL-4, and pools of puromycin-resistant cells were generated with each knockdown vector, as previously described [39,46].

**Figure 6:**
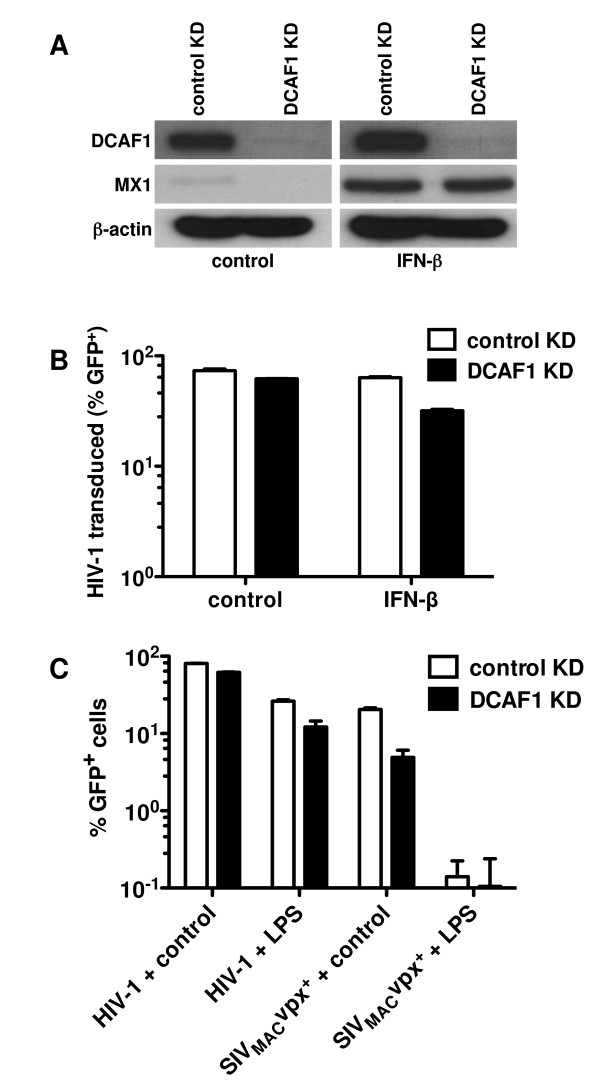
**DCAF1 (VPRBP) knockdown does not prevent Vpx rescue of HIV-1 from the antiviral state in MDDCs**. MDDCs were transduced with lentiviral knockdown vectors targeting either DCAF1, or a control RNA, in the presence of SIV VLPs. DCAF1 KD and control KD cells were then treated with IFN-β for 24 hrs, and lysates were probed in immunoblots with antibodies against the indicated proteins (**A**), or cells were challenged with a VSV-G-pseudotyped HIV-1_NL4-3 _GFP reporter virus (**B**). (**C**) DCAF1 KD and control KD MDDCs were treated with LPS for 24 h, and challenged with either VSV-G-pseudotyped HIV-1_NL4-3 _or SIV_MAC-239 _GFP reporter viruses. Data represent one of at least three independent experiments. Error bars represent ± SD (n = 3).

Lysate from MDDCs that had been transduced with knockdown vector targeting DCAF1 was examined by Western blot. In contrast to the strong signal observed with the control knockdown cells, DCAF1 protein was undetectable in the DCAF1-knockdown cells (Figure 6A), even after cells had been treated with exogenous IFN-β. The ability of the cells to respond to IFN-β was confirmed by showing the induction of Mx1 protein (Figure 6A). Despite this highly efficient DCAF1 knockdown, little change was observed in the ability of Vpx to rescue HIV-1 transduction from the antiviral state established by IFN-β or by LPS (Figure 6B and 6C). Parallel experiments in MDDCs from the same donor showed that transduction with SIV_MAC _was efficiently blocked by IFN-β or by LPS (Figure 6C), demonstrating that the antiviral state had been well-established in these cells.

### The IFN-specific, *vpx*-sensitive block to HIV-1 is in the MDDC nucleus

**Figure 7:**
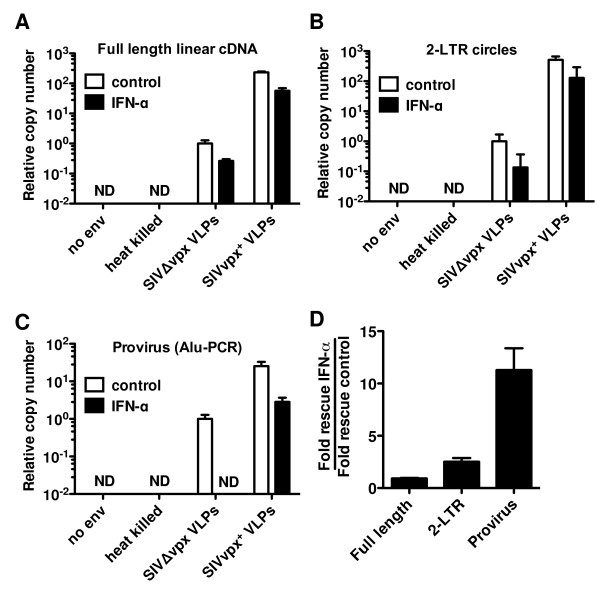
**SIV_MAC _Vpx rescues HIV-1 from the antiviral state in MDDC prior to establishment of the provirus**. MDDCs were treated with IFN-α for 24 h, and then treated with SIV_MAC _VLPs or media for 3 h, and finally challenged with a VSV-G-pseudotyped HIV-1_NL4-3 _GFP reporter virus. Total DNA was extracted from 5 × 10^6 ^MDDCs and qPCR was performed for HIV-1 full-length linear reverse transcription products (**A**), 2-LTR circles (**B**), and provirus (**C**). (**D**) Data from **A**, **B**, **C**, represented as (fold-rescue of HIV-1 by Vpx from IFN-α treatment) divided by (fold-rescue of HIV-1 by Vpx in the absence of exogenous IFN). Data represent one of at least three independent experiments. Error bars represent ± SEM (n = 4).

Vpx is required for the synthesis of SIV_MAC _or HIV-2 cDNA after infection of MDDCs or MDMs [10,13,14]. VLPs bearing Vpx similarly increase the levels of nascent HIV-1 cDNA after infection of these cell types [10]. In the absence of exogenous IFN, Vpx^+ ^VLPs indeed increased the levels of full-length linear HIV-1 cDNA (Figure 7A). The increase in the levels of 2-LTR circles (Figure 7B) and Alu-PCR products (Figure 7C) were of comparable magnitude. Heat-inactivated virus and virions generated in the absence of Env were used as controls to demonstrate that the PCR products were a reflection of *de novo *cDNA synthesis in the target cells and were not the result of contaminating plasmid DNA carried over from the transfection used to generate the viruses. These experiments indicate that, in the absence of exogenous IFN, the main effect of Vpx is to increase the efficiency of HIV-1 reverse transcription.

When MDDCs were treated with IFN-α prior to challenge with HIV-1, the magnitude rescue of full-length viral cDNA and 2-LTR circles by Vpx was identical to the magnitude rescue by Vpx in the absence of exogenous IFN (Figure 7D). In contrast, the magnitude rescue of proviral DNA by Vpx was at least 12-fold greater when MDDCs had been treated with exogenous IFN than with untreated MDDCs (Figure 7C and 7D). The magnitude of this rescue possibly underestimates the real difference, since the Alu-PCR signal was below the limit of detection when DNA from IFN-treated cells was used as template, even after 50 cycles of amplification. These data indicate that the IFN-specific effect of Vpx in MDDCs occurs after the preintegration complex is transported to the MDDC nucleus.

## Conclusions

The experiments presented here demonstrate that SIV_MAC_/HIV-2 Vpx rescues HIV-1 from the antiviral state established by exogenous type I IFN or LPS in MDDCs. This phenotype is truly extraordinary in that Vpx offered complete rescue of HIV-1, after the antiviral state had been fully established, and the magnitude of the rescue approached 1000-fold. Surprisingly, the presence of Vpx in SIV_MAC _or HIV-2 did not protect these viruses from IFN-β or LPS treatment, even when target cell MDDCs were treated with VLPs bearing additional Vpx prior to challenge with reporter virus. Although Vpx is not normally an HIV-1 accessory protein, it provides a powerful tool that will aid attempts to identify new HIV-1 restriction factors that are elicited by IFN in dendritic cells.

Elucidation of the mechanism by which Vpx rescues HIV-1 from the antiviral state would be aided enormously by an experimental system that exploits a cell line. Among cell lines tested, the most pronounced phenotype was observed with the acute monocytic leukemia cell line THP-1 [47], which had been treated with phorbol esters to promote differentiation into macrophages, as we reported previously to study Vpx and innate immune signaling [11,39]. The magnitude inhibition of HIV-1 transduction by LPS or IFN-β in THP-1 macrophages [11,39] was 10-fold less than that seen in MDDC. Of greater concern, though, rescue of HIV-1 from the antiviral state by Vpx^+ ^VLPs in these cells was only 2 to 10-fold (data not shown). Ongoing mechanistic studies concerning the Vpx phenotype reported here, then, will likely not be possible with a cell line.

HIV-1 transduction of monocyte-derived macrophages (MDMs) was also greatly stimulated by Vpx; although, in the absence of exogenous IFN, HIV-1 transduction efficiency was lower in these cells than in MDDCs (data not shown). A necessary consequence is that a smaller proportion of the Vpx effect in MDMs was specific to the antiviral state. In other words, the magnitude rescue of HIV-1 by Vpx following establishment of the antiviral state with exogenous IFN was most evident in MDDCs. In the presence of exogenous type 1 IFN, MDDCs might express an HIV-1-specific, Vpx-sensitive, anti-viral effector at higher levels than do MDMs. Alternatively, constitutive expression levels of this putative factor might be higher in MDMs.

Viruses often encode factors that prevent establishment of the antiviral state. For example, hepatitis C virus, poliovirus, and rhinovirus proteases degrade MDA-5, RIG-I, IPS-1, and TRIF [48-53]. In the experimental system reported here, Vpx was administered after the antiviral state was fully established. Therefore, Vpx does not act by blocking induction of the antiviral state. This is consistent with the observation that *vpx *had no significant effect on the transcriptional induction of luciferase reporters for critical innate immune factors, including IFN-β, NF-κB, or AP-1 (additional file 4, Figure S4).

Additionally, Vpx appears not to launch a global shutdown of the antiviral state. It caused no change in levels of MDDC cell surface markers for maturation, in IFN-β secretion and steady-state protein levels for MX1 and APOBEC3A, or in steady-state levels of mRNAs produced by 8 ISGs (additional file 1, Figure S1). More importantly, Vpx did not rescue SIV_MAC _or HIV-2, indicating that the antiviral state was very much intact following exposure to Vpx. More likely, Vpx inactivates an HIV-1-specific antiviral effector that is induced by IFN. This inactivation might involve degradation, the same way that Vif promotes the degradation of APOBEC3G [30-32] or Vpu promotes the degradation of TETHERIN [54-56]. Alternatively, Vpx might sequester the putative factor, blocking it without assistance from ubiquitination machinery, as may also be the case with Vif and Vpu [57,58].

Though it has been known for over 20 years that type 1 IFN and LPS block HIV-1 infection of myeloid cells [40], the effector proteins responsible for the block to HIV-1 transduction of IFN-treated MDDCs is not known. Several ISG-encoded proteins inhibit HIV-1, APOBEC3G [59] and Tetherin [60,61] being prominent among them. These host restriction factors pose obstacles to infection of sufficient importance that HIV-1 maintains two of its nine genes - *vif *and *vpu*, respectively - to counteract them. Neither Vif nor Vpu is required for the phenotype reported here since Vpx rescued minimal HIV-1 vectors lacking all viral accessory proteins as efficiently as it rescued full HIV-1 virus. Additionally, the best-characterized phenotypes of Vif and Vpu require their presence during virion assembly and the experiments reported here likely involve effects of Vpx that are restricted to the target cell.

TRIM5, another restriction factor encoded by an ISG, is required for establishment of an antiviral state by LPS in MDDCs [39]. Nonetheless, endogenous human TRIM5 is unlikely to be a direct antiviral effector in the experiments reported here since inhibition of HIV-1 transduction by exogenous type 1 IFN is not reversed by TRIM5 knockdown [39]. Other TRIM proteins are encoded by ISGs [62], and some of these exhibit antiviral activity [63]. TRIM22, for example, blocks HIV-1 LTR-directed transcription [64], but the putative antiviral effector in IFN-treated MDDCs acts before integration, as documented by Alu-PCR (Figure 7). Additionally, TRIM22 does not block transcription from the heterologous promoter (SFFVp) used in the transduction vectors here [64].

In the course of examining ISG expression levels in MDDCs it was observed that, in response to exogenous type 1 IFN or LPS, APOBEC3A mRNA levels increased nearly 10,000-fold and the protein levels also increased to an impressive extent (additional file 1, Figure S1C). APOBEC3A is a nuclear protein [65,66] and therefore a reasonable candidate for the Vpx-sensitive, IFN-stimulated, anti-HIV-1 effector protein. Specific association of APOBEC3A with Vpx was not detected in co-transfection experiments in 293T cells, and no effect on inhibition of HIV-1 was observed when APOBEC3A knockdown was attempted with lentiviral vectors or with transfected double-stranded RNA oligonucleotides (data not shown). These findings are in contrast to reports that Vpx associates with APOBEC3A and that a *vpx *mutant that does not bind to APOBEC3A failed to stimulate HIV-1 infection of monocytes [67]. APOBEC3A knockdown was also reported to render monocytes more permissive for HIV-1 [68]. These discrepancies with the results reported here might be due to cell type differences, i.e, monocytes versus MDDCs, or other differences in methodology.

Vpx was recently shown to bind to SAMHD1 and promote the degradation of this myeloid cell protein [69,70]. While SAMHD1 is clearly a Vpx-sensitive inhibitor of HIV-1 replication in myeloid cells, it does not appear to be the IFN-stimulated HIV-1 inhibitor described here. SAMHD1 knockdown in THP-1 cells results in more than 10-fold increase in HIV-1 replication [70]; in contrast to the enormous effect of Vpx in IFN-treated MDDCs, HIV-1 infection of IFN-treated THP-1 cells increases only two to three-fold in response to Vpx.

Both Vpr and Vpx bind DCAF1 (VPRBP) and associate with the DDB1/RBX1/CUL4A E3 ubiquitin ligase complex [12,13,15,33-37,71,72]. Vpr might, therefore, be expected to interfere with Vpx binding to DCAF1 and the E3 complex. However, the presence of HIV-1 Vpr or SIV_MAC _Vpr did not significantly alter the ability of SIV_MAC _Vpx to protect HIV-1 from the antiviral state, underlying the unique ability of Vpx to protect HIV-1.

The unexpected finding that Vpx mutant proteins that do not bind to DCAF1 (Figure 5A and references [12,13,15,35]) retain the ability to rescue HIV-1 from exogenous IFN indicates that the DCAF1/DDB1/RBX1/CUL4A E3 ubiquitin ligase complex is dispensable for the phenotype reported here. Consistent with this result was the demonstration that Vpx rescued HIV-1 in the presence of an effective DCAF1 knockdown (Figure 6). While the DCAF1/DDB1/RBX1/CUL4A E3 ubiquitin ligase complex, and Vpx, is clearly required for SIV_MAC _to infect human macrophages in the absence of exogenous type 1 IFN [12], Vpx interaction with DCAF1 was also not required for HIV-1 transduction of THP-1 macrophages [11]. These results indicate that, if Vpx rescues HIV-1 from the antiviral state by promoting the degradation of an antiviral effector, it does so by recruiting a yet-to-be-identified E3 ubiquitin ligase complex.

As previously reported [10,13,14], Vpx had a large effect on HIV-1 reverse transcription in transduced MDDCs (Figure 7). An additional effect of Vpx was observed, though, that was specific to the cells that had been treated with exogenous type 1 IFN: Vpx overcame a block to HIV-1 transduction that occurred after the virus had entered the target cell nucleus (Figure 7). Thus, it may be that Vpx protects HIV-1 from more than one antiviral factor. The first factor is constitutively expressed in myeloid cells and blocks reverse transcription. The second factor is induced by IFN and acts in the nucleus to block transduction. HIV-1 CA and IN, two proteins essential at this stage of the HIV-1 replication cycle [28,73,74], would be likely targets of this antiviral factor. To date, attempts to demonstrate the importance of these proteins by transferring Vpx-responsiveness using chimeric viruses have not been successful due to the poor infectivity of these constructs in highly permissive cell lines, let alone in MDDCs.

Why does Vpx protect HIV-1, and not SIV_MAC _or HIV-2, from the antiviral state in MDDCs? A number of scenarios are possible. It might be that there is an IFN-inducible, HIV-1-specific inhibitor, which is suppressed by Vpx. This factor might be induced by the recently reported HIV-1-specific, cryptic sensor in MDDCs [75]. In this case, one would need to invoke an additional, IFN-induced, SIV_MAC_-specific factor, which is not suppressed by Vpx. Alternatively, there might be a single IFN-induced inhibitor of both viruses, from which Vpx offers protection to HIV-1 but not to SIV_MAC_. Whichever scenario is correct, identification of antiviral factors such as these has the potential to guide development of new drugs for inhibiting HIV-1 replication in the clinical context. Additionally, given the critical role of dendritic cells at the interface between the innate and acquired immune systems [76,77], identification of such factors may aid attempts to understand how the innate immune system detects HIV-1, and assist efforts to stimulate acquired immune responses to HIV-1 [39,78].

## Methods

### Ethics statement

Buffy-coats obtained from anonymous blood donors were provided by the Blood Transfusion Center of the Hematology Service of the University Hospital of Geneva by agreement with the Service, after approval of our project by Ethics Committee of the University Hospital of Geneva (Ref# 0704).

### Chemicals and drugs

The following compounds were used at the given final concentrations: Ultrapure LPS from *E. coli *K12 (100 ng/mL), poly(I:C) (25 μg/mL, or 2 μg/mL when complexed with Lipofectamine 2000 (Invitrogen)), and poly(dA:dT) (2 μg/mL) were obtained from Invivogen. Recombinant, human IFN-β (10 ng/mL) and recombinant, human IFN-α2a (10 ng/mL) were obtained from PBL InterferonSource. All other chemicals and drugs were obtained from Sigma-Aldrich, unless otherwise noted.

### Cell lines and primary cell cultures

HEK-293 and 293T cells were obtained from American Type Culture Collection (ATCC) and were grown in Dulbecco's modified Eagle medium (D-MEM) (high glucose) (Invitrogen) supplemented with 10% fetal bovine serum (FBS) (Hyclone), 1 × MEM Non-Essential Amino Acids (NEAA) Solution (Invitrogen), and 1 × GlutaMAX-I (Invitrogen). 293T cells were periodically grown in cell culture medium containing 500 μg/mL Geneticin (Invitrogen) to maintain expression of the SV40 large T antigen.

THP-1 cells were obtained from ATCC and maintained in RPMI-1640 (Invitrogen) supplemented with 10% FBS, 20 mmol/L HEPES (Invitrogen), 1 × MEM NEAA, and 1 × GlutaMAX-I. In order to differentiate THP-1 monocytes into macrophage-like cells, THP-1 cells were counted, centrifuged at 200 × *g *for 10 min, and resuspended at a concentration of 1 × 10^6 ^cells/mL in fresh cell culture medium containing 100 ng/mL phorbol 12-myristate 13-acetate (PMA). Cells were plated into each well of a sterile tissue culture plate (2 mL culture/well of a 6-well plate or 200 μL culture/well of a 96-well flat-bottom plate) and allowed to differentiate for 24 h, at which point the PMA-containing medium was removed and fresh cell culture medium (without PMA) was added. The cells were rested for an additional 48 h before use.

Peripheral blood mononuclear cells (PBMCs) were isolated from buffy coats prepared from healthy, anonymous donors using Ficoll-Paque Plus (GE Healthcare) following the protocol supplied by Miltenyi Biotec. CD14^+ ^cells (monocytes) were enriched from PBMCs by positive selection using CD14 MicroBeads (Miltenyi Biotec) with purity routinely greater than 95%, as determined by flow cytometry after staining with PE anti-human CD14 (BD Biosciences). Enriched CD14^+ ^cells were counted, centrifuged at 200 × *g *for 10 min, and resuspended in RPMI-1640 supplemented with 10% FBS, 20 mmol/L HEPES, 1 × MEM NEAA, and 1 × GlutaMAX-I, at a concentration of 1 × 10^6 ^cells/mL. In order to generate monocyte-derived macrophages (MDM), recombinant, human GM-CSF (R&D Systems) was added to the cell suspension to a final concentration of 50 ng/mL, and in order to generate monocyte-derived dendritic cells (MDDC), recombinant, human IL-4 (R&D Systems) was added to a final concentration of 25 ng/mL along with 50 ng/mL GM-CSF. CD14^+ ^cells were allowed to either differentiate into MDDCs in the presence of GM-CSF and IL-4 for 4 d, or into MDMs in the presence of GM-CSF alone for 10 d, before use. The following antibodies were used for flow cytometry: APC anti-CD86 (BU63) was from EXBIO; FITC anti-CD1a (HI149), PE anti-CD209 (DC-SIGN) (DCN46), and APC-anti-CD83 (HB15e) were from BD Biosciences. Isotype controls were from Miltenyi Biotec.

All primary cells and cultured cell lines were maintained in cell culture media without penicillin or streptomycin, and were cultured at 37°C in a humidified incubator containing 5% carbon dioxide.

### Plasmids, Vectors, and Viruses

SIV_MAC-251 _*vpx*, HIV-2_ROD _*vpx*, SIV_SMM-PBj _*vpx*, and SIV_AGM-TAN _*vpr *were codon-optimized for expression in human cells using services provided by Sloning BioTechnology GmbH (Puchheim, Germany). See additional file 5, Table S1 for the codon-optimized nucleic acid sequences. The codon optimized cDNAs were cloned into pcDNA3.1(-) (Invitrogen) by PCR using the primer pairs listed in additional file 6, Table S2. Alanine substitution mutations were introduced into the codon-optimized SIV_MAC-251 _*vpx *cDNA by overlapping PCR, using the primer sets detailed in additional file 6, Table S2. APOBEC3A, APOBEC3A:Myc:6 × His, APOBEC3G, and APOBEC3G:Myc:6 × His expression constructs were provided by Dr. Klaus Strebel (National Institute of Allergy and Infectious Diseases, NIH). FLAG:HA:AU1:DCAF1 and FLAG:HA:AU1:DDB1 expression constructs were provided by Dr. Jacek Skowronski (Case Western Reserve University).

pFSGW, an HIV-1-based transfer vector with EGFP expression under the control of the spleen focus-forming virus (SFFV) long terminal repeat (LTR), as well as gag-pol and VSV G expression plasmids, are described elsewhere [39]. pSIV3+, a SIV_MAC-251 _gag-pol expression plasmid [79], and pSIV3+Δ*vpx*, generated by digest with BstB1 and religation after blunting ends with DNA Polymerase I, Large (Klenow) Fragment (New England BioLabs), introducing a premature stop codon at amino acid 25 of *vpx*, were provided by Dr. Andrea Cimarelli (École Normale Supérieure de Lyon). pNL4-3 Nef_NA7_:GFP (CCR5-tropic), which bears the V3 loop of the CCR5-tropic 92TH014-2 HIV-1 strain and where Nef_NA7 _is fused to EGFP [80,81]. pNL4-3.GFP.E- [82] and pNL4-3.Luc.E- [83] are pNL4-3 with an env^- ^inactivating mutation and EGFP or luciferase, respectively, cloned in place of *nef*. The HIV-2 and HIV-2Δ*vpx *packaging plasmids, as well as the HIV-2 GFP transfer vector, are described elsewhere [10]. p8.9NDSB is a minimal HIV-1 packaging plasmid [84]. The SIV_MAC _Vpx binding motif (DPAVDLL) was generated and introduced into HIV-1 Gag p6 by overlapping PCR and cloned into the BglII and BclI sites of p8.9NDSB using the following primers: p6 BglII 5': 5'-TAGGGAAGATCTGGCCTTCCCACAA-3', p6 Vpx ins 3': 5'-TAGCAGATCCACAGCTGGGTCTTCTGGTGGGGCTGTTGGCTCTGG-3', p6 Vpx ins 5': 5'-GACCCAGCTGTGGATCTGCTAGAGAGCTTCAGGTTTGGGGAAGA-3', p6 BclI 3': 5'-ATGAGTATCTGATCATACTGTCTTACTT-3'. SIV_MAC-239 _env^- ^GFP is described elsewhere [85]. psSIV-GAE is pSIV-GAE [86], a SIV_MAC-251 _transfer vector expressing GFP, where the cytomegalovirus (CMV) promoter driving EGFP expression was replaced with the SFFV LTR, amplified by PCR from pFSGW.

### Production of viruses, vectors, and virus-like particles (VLPs)

Viruses, minimal vectors, and VLPs were produced by transfection of 293T cells using Lipofectamine 2000 (Invitrogen), according to the manufacturer's instructions. For three-part vector systems, the following DNA ratio was used: 4 parts transfer vector: 3 parts packaging plasmid: 1 part envelope. For two-part virus systems a 7:1 ratio was used (7 parts env^- ^virus: 1 part envelope). For VLPs, a 7:1 ratio was used (7 parts gag-pol expression plasmid: 1 part envelope). 16 h after transfection the transfection medium was replaced with fresh target-cell medium. 48 h after transfection the supernatant was collected, centrifuged at 200 × *g *for 5 min, filtered through a sterile 0.45 μm syringe filter (Millipore), and stored in 1 mL aliquots at -80°C. When comparing viruses, vectors, or VLPs, samples were normalized by single-cycle infectivity assays on HEK-293 cells and/or the reverse transcriptase (RT) activity present in the viral supernatant by qRT-PCR [87].

### RNAi in primary human monocyte-derived dendritic cells and macrophages

To generate stable microRNA-based shRNA knockdowns in primary human MDDC or MDM, human CD14^+ ^cells, freshly isolated from PBMC as described above, were treated with SIV_MAC-251 _VLPs for 3 h, and then transduced with either a control or experimental pAPM microRNA-based shRNA vectors [39]. The CD14^+ ^cells were then allowed to differentiate into MDDC or MDM as described above. After differentiation, the MDDC or MDM were selected with 10 μg/mL puromycin dihydrochloride for 3 d and assayed for knockdown by SDS-PAGE/western blot and/or qRT-PCR analysis. Using this technique we routinely observed greater than 90% transduced MDDC or MDM. shRNA target sequences used: *Luciferase*: 5'-TACAAACGCTCTCATCGACAAG-3', *APOBEC3A *5'-TTGGCTTCATATCTAGACTAAC-3', *DCAF1 *(*VPRBP*): 5'-AGCACTTCAGATTATCATCAAT-3'.

For transient siRNA in MDM, 5 × 10^5 ^cells were plated in each well of a 6-well plate. 600 pmol of ON-TARGETplus siRNA Smart pools (Thermo Scientific Dharmacon) targeting *DCAF1*, *APOBEC3A*, or control siRNA were complexed with 5 μL Lipofectamine 2000 (Invitrogen), following the manufacturer's instructions, and added to 2 mL of cell culture media. The transfection medium was removed after 6 h and replaced with fresh cell culture medium. Knockdown was assessed by SDS-PAGE/western blot and/or qRT-PCR analysis 48-72 h after transfection. For transient siRNA in MDDC, 8 × 10^5 ^were plated in each well of a 12-well plate in 600 μL culture medium. 100 nM of FlexiTube GeneSolutions pooled siRNAs (Qiagen) targeting *DCAF1*, *APOBEC3A*, or control siRNA were complexed with 6 μL of HiPerFect Transfection Reagent (Qiagen), following the manufacturer's instructions, and added to the MDDC culture. A second round of transfection was performed 24 h later. Knockdown was assessed by SDS-PAGE/western blot and/or qRT-PCR analysis 24-48 h after the second transfection.

### Quantitative reverse transcription polymerase chain reaction (qRT-PCR)

Total RNA was extracted from 2 × 10^6 ^cells using the RNeasy Plus Mini Kit (Qiagen), following the manufacturer's instructions. First-strand cDNA was generated using the SuperScript III First-Strand Synthesis System (Invitrogen) using 2 μg total RNA and random hexamers, according to the manufacturer's protocol. The qPCR reaction was performed in triplicate in a 20 μL volume using 1 × TaqMan Gene Expression Master Mix (Applied Biosystems), 1 μL of undiluted cDNA, and 1 μL of the specified TaqMan Gene Expression Assay (Applied Biosystems) on either the 7900HT Fast Real-Time PCR System (Applied Biosystems) or the CFX96 Real Time System/C1000 Thermal Cycler (Bio-Rad) using the following program: 95°C for 10 min and 45 cycles of 95°C for 15 s and 60°C for 1 min. The following TaqMan Gene Expression Assays were used: *APOBEC3A *(Hs00377444_m1), *APOBEC3G *(Hs00222415_m1), *CCL2 *(Hs00234140_m1), *CCL8 *(Hs00271615_m1), *CUL4A *(Hs00757716_m1), *CXCL10 *(Hs00171042_m1), *DDB1 *(Hs00172410_m1), *IFIT1 *(Hs00356631_g1), *IFIT2 *(Hs00533665_m1), *IFNB1 *(Hs01077958_s1), *IL6 *(Hs00985639_m1), *MX1 *(Hs00182073_m1), *PTGS2 *(Hs00153133_m1), *TNF *(Hs00174128_m1), *TRIM5 *(Hs01552558_m1), and *VPRBP *(Hs00206762_m1). *OAZ1 *(Hs00427923_m1) was used as an endogenous control. Data were analyzed using the SDS software v2.2.2 (Applied Biosystems) or the CFX Manager software v1.6 (Bio-Rad), which calculate relative mRNA expression levels using the standard comparative Ct method (2^-ΔΔCt^) with error bars representing ± the standard error of the mean.

### Sodium dodecyl sulfate polyacrylamide gel electrophoresis (SDS-PAGE)/western blot analysis

2 × 10^6 ^cells were lysed in 200 μL 1 × Laemmli sample buffer (62.5 mmol/L Tris, pH 6.8, 2% SDS, 10% glycerol, 357.5 mmol/L 2-mercaptoethanol (2-ME), 0.0025% bromophenol blue (Bio-Rad)), supplemented with 1 × Halt Protease and Phosphatase Inhibitor Cocktail, EDTA-free (Thermo Fisher Scientific) and 5 mmol/L EDTA, pH 8.0. Whole-cell lysates were heated at 100°C for 5 min, centrifuged at 14, 000 × *g *for 2 min, and 50 μL were loaded onto a 10% ProSieve 50 gel (Lonza) for SDS-PAGE. Relative protein concentration was normalized after staining the gel with SimplyBlue SafeStain (Invitrogen). After SDS-PAGE, proteins were transferred onto an Immun-Blot polyvinylidene fluoride (PVDF) membrane (Bio-Rad) for 16 h at 30 V (constant voltage) at 4°C. After the protein transfer, the membrane was washed 3 × 5 min with deionized water and blocked 1 h at 4°C with 5% non-fat dry milk dissolved in TBST (Tris-buffered saline (50 mmol/L Tris, pH 7.4, 150 mmol/L sodium chloride (NaCl)) with 0.1% Polysorbate 20 (Tween 20)). The membrane was then washed 3 × 5 min with TBST. The primary antibody was diluted to 1 μg/mL in 5% non-fat dry milk dissolved in TBST and added to the membrane and incubated 16 h at 4°C with gentle rocking. After the 16 h incubation with the primary antibody the membrane was washed 3 × 5 min with TBST. The secondary antibody was diluted 1:10,000 in 5% non-fat dry milk dissolved in TBST and added to the washed membrane and incubated at room temperature for 1 h with gentle rocking. The membrane was washed 3 × 10 min and developed using either the ECL or ECL Plus Western Blotting Detection Reagents (GE Healthcare Life Sciences) and exposed to Hyperfilm ECL film (GE Healthcare Life Sciences).

The following antibodies were used in this study: rabbit anti-VPRBP (DCAF1) and rabbit anti-MX1 were from Proteintech Group; rabbit anti-APOBEC3A/APOBEC3G serum was a generous gift from Dr. Klaus Strebel (National Institute of Allergy and Infectious Diseases, NIH); mouse anti-FLAG (M2), rabbit anti-FLAG, mouse anti c-Myc (9E10), rabbit anti-c-Myc, mouse anti-HA (HA-7), rabbit anti-HA, and mouse anti-β-Actin (AC-74) were from Sigma-Aldrich. The following reagents were obtained through the AIDS Research and Reference Reagent Program, Division of AIDS, NIAID, NIH: monoclonal antibody to HIV-1 p24 (SIV_MAC _p27) (AG3.0) [88] from Dr. Jonathan Allan and HIV-2 Vpx Monoclonal Antibody (6D2.6) [89] from Dr. John C. Kappes. Secondary antibodies used: HRP-linked donkey anti-rabbit IgG and HRP-linked sheep anti-mouse IgG were from GE Healthcare Life Sciences.

### Immunoprecipitation (IP)

Sub-confluent 293T cells grown on T-75 tissue culture flasks were transfected with 16 μg total plasmid DNA using TransIT-LT1 Transfection Reagent (Mirus Bio), following the manufacturer's protocol. 40 h post-transfection, cells were washed with 30 mL ice-cold phosphate buffered saline (PBS) (Invitrogen) and lysed with 1 mL ice-cold lysis buffer (50 mmol/L Tris, pH 7.4, 150 mmol/L NaCl, 0.5% CHAPSO (Affymetrix)) supplemented with 1 × Halt Protease and Phosphatase Inhibitor Cocktail, EDTA-free (Thermo Fisher Scientific). Crude cell lysates were scraped off the surface of the flask, transferred to pre-chilled 2 mL microcentrifuge tubes, and rotated at 4°C for 20 min. The crude cell lysates were then centrifuged at 14,000 × *g *for 10 min and 900 μL of the clarified lysate was transferred to pre-chilled microcentrifuge tubes, and the remaining 100 μL of the clarified lysate was diluted equally in 2 × Laemmli sample buffer, incubated at 100°C for 5 min, and stored at -80°C. 2 μg antibody was conjugated to 50 μL of Protein G Dynabeads (Invitrogen) following the manufacturer's protocol. The beads were washed 3 times with 1 mL ice-cold lysis buffer, resuspended in 100 μL lysis buffer, and added to the clarified cell lysates. After 2 h rotating at 4°C, the Protein G magnetic bead immune complexes were washed 4 times with 1 mL ice-cold lysis buffer, resuspended in 100 μL of 1 × Laemmli sample buffer, incubated at 100°C for 5 min, and stored at -80°C until ready for SDS-PAGE.

### Vpx incorporation assay

Using TransIT-LT1 Transfection Reagent (Mirus Bio), sub-confluent 293T cells grown on T-75 flasks were transfected with the following plasmids: for SIV_MAC-251 _Vpx incorporation in HIV-1, 14 μg p8.9NDSB (containing HIV-1 Gag WT) or p8.9NDSB-DPAVDLL (containing HIV-1 Gag with the SIV_MAC _Vpx binding motif inserted into p6) along with 2 μg empty pcDNA3.1(-) or pcDNA3.1(-) expressing codon-optimized SIV_MAC-251 _*vpx *WT; for SIV_MAC-251 _Vpx incorporation into SIV_MAC-251_, 14 μg pSIV3+Δ*vpx *and 2 μg empty pcDNA3.1(-) or pcDNA3.1(-) expressing codon-optimized SIV_MAC-251 _*vpx *WT or the indicated mutants. 40 h after transfection, the medium was collected, clarified by centrifugation for 10 min at 500 × *g*, and filtered through a sterile 0.45 μm syringe filter (Millipore). The supernatant was then overlaid on 8 mL of TNE buffer (50 mmol/L Tris pH 7.5, 140 mmol/L NaCl, 5 mmol/L EDTA, pH 8.0) containing 25% (w/v) sucrose in a 30 mL Beckman Coulter ultracentrifuge tube, and subjected to 26,000 rpm for 90 min. The supernatant was then removed and the pellet was dissolved in 1 × Laemmli sample buffer, heated at 100°C for 5 min, and stored at -80°C until SDS-PAGE.

### Luciferase assays

HEK-293 cells were plated on white, opaque 96-well CulturPlate-96 microplates (PerkinElmer) at a concentration of 2 × 10^4 ^cells per 100 μL tissue culture medium per well, 24 h prior to transfection. Cells were transfected with Lipofectamine 2000, using 0.5 μL Lipofectamine 2000 per well, with 1 ng of the Renilla luciferase internal control reporter plasmid pRL-TK (Promega), 5 ng firefly luciferase experimental reporter plasmid, and 15 to 50 ng of pcDNA3.1(-) containing the experimental cDNA or empty pcDNA3.1(-) as a control, following the manufacturer's instructions. Each experimental condition was performed in sextuplicate. 48 h after transfection, the plate was assayed using the Dual-Glo Luciferase Assay System (Promega) and read using a Veritas Microplate Luminometer (Turner Biosystems). Firefly luciferase readings were normalized to Renilla luciferase readings in each well, and the data are represented as fold-change compared to empty pcDNA3.1(-) (± the standard deviation). The following firefly luciferase plasmids were used: the *IFNB1*-luc construct was a gift from Dr. Jürg Tschopp (University of Lausanne); the *Prl *promoter AP-1-luc construct was from Dr. Ruslan Medzhitov (Yale School of Medicine), and the NF-κB-luc construct was from Dr. Jurgen Brojatsch (Albert Einstein College of Medicine). The MAVS, MYD88, and TAB3 expression constructs are described elsewhere [39].

For single-cycle infectivity assays using the env^- ^luciferase reporter viruses described above, HEK-293 cells or THP-1 macrophages were plated on white, opaque 96-well CulturPlate-96 microplates (PerkinElmer) at 1 × 10^5 ^cells per well in 100 μL tissue culture medium. After 24 h, the cells were challenged with serial dilutions of luciferase reporter viruses. 72 h after transduction the plate was assayed using the Bright-Glo Luciferase Assay System (Promega) and read using a Veritas Microplate Luminometer (Turner Biosystems).

### IFN-β secretion assay

IFN-β secretion was quantified using the reporter cell line HL116, which carries the luciferase gene under the control of the IFN-inducible 6-16 promoter [90] (a kind gift from Dr. Olivier Schwartz, (Institut Pasteur)). HL116 cells, grown in DMEM supplemented with 10% FBS and hypoxanthine-aminopterin-thymidine (HAT) media supplement, were plated at 2 × 10^4 ^cells per well of a 96-well plate, 16 h prior to the assay. The HL116 cells were then incubated for 7 h with the culture medium from MDDC challenged with SIV_MAC-251 _VLPs or a control for 3 h, and then treated with LPS. A titration of recombinant, human IFN-β (PBL InterferonSource) was used as a standard. Cells were then lysed with Luciferase Cell Culture Lysis 5 × Reagent (Promega) and luciferase activity was measured using the Luciferase Assay Reagent (Promega), following the manufacturer's instructions.

### qPCR for viral cDNA

qPCR for HIV-1 full-length linear cDNA and 2-LTR circles was performed as described previously [91,92], with the following modifications. The qPCR reaction was performed in quadruplicate in a 20 μL volume using 1 × TaqMan Universal PCR Master Mix (Applied Biosystems), 500 ng of total cellular DNA (isolated from 5 × 10^6 ^MDDC using the DNeasy Blood & Tissue Kit (Qiagen)), and 300 nmol/L forward primer, 300 nmol/L reverse primer, and 100 nmol/L probe. Using the 7900 HT Fast Real-Time PCR System (Applied Biosystems), the following program was performed: 50°C for 2 min, 95°C for 10 min, then 40 cycles of 95°C for 15 s and 60°C for 1 min. The following primer and probe sets were used: Late RT: J1 5'-ACAAGCTAGTACCAGTTGAGCCAGATAAG-3', J2 5'- GCCGTGCGCGCTTCAGCAAGC-3', Late RT probe (LRT-P) 5'- (FAM)-CAGTGGCGCCCGAACAGGGA-(TAMRA)-3'; 2-LTR circles: 2-LTR forward (MH535): 5'-AACTAGGGAACCCACTGCTTAAG-3', 2-LTR reverse (MH536) 5'-TCCACAGATCAAGGATATCTTGTC-3', 2-LTR probe (MH603) 5'-(FAM)-ACACTACTTGAAGCACTCAAGGCAAGCTTT-(TAMRA) -3'.

Nested Alu-LTR qPCR for HIV-1 provirus was performed as described previously [91,93] with the following modifications. Total DNA was prepared from 5 × 10^6 ^cells using the DNeasy Blood and Tissue Kit (Qiagen). 500 ng total DNA was diluted in a 50 μL reaction with 1 × AccuPrime PCR Buffer II (Invitrogen), 0.2 μL AccuPrime High Fidelity Taq polymerase (Invitrogen), and 400 nmol/L of each primer (Alu forward (MH535) 5'-AACTAGGGAACCCACTGCTTAAG-3' and Alu reverse (SB704) 5'-TGCTGGGATTACAGGCGTGAG-3'). After 2 min denaturing at 94°C, 25 cycles of 94°C for 15 s, 55°C for 30 s, and 68°C for 3 min were performed, followed by a 10 min extension at 68°C. The second round of amplification using a nested primer/probe set was performed in quadruplicate in a 20 μL volume using 1 × TaqMan Universal PCR Master Mix (Applied Biosystems), 2 μL of the first round PCR as template, 300 nmol/L of each forward and reverse primer, and 100 nmol/L probe on the 7900HT Fast Real-Time PCR System (Applied Biosystems) using the standard mode program with the following changes: 50°C for 2 min, 95°C for 10 min, and 50 cycles of 95°C for 15 s and 60°C for 90 s. The following primer/probe combinations were used: Alu II forward 5'-GGTAACTAGAGATCCCTCAGACCCT-3', Alu II reverse 5'-GCGTGAGCCACCGC-3', Alu II probe 5'-(FAM)-TTAGTCAGTGTGGAAAATCTCTAGCAGGCCG-(TAMRA)-3'.

Mitochondrial DNA was used as an internal control with the following primer/probe set: Mito forward (MH533) 5'-ACCCACTCCCTCTTAGCCAATATT-3', Mito reverse (MH534) 5'-GTAGGGCTAGGCCCACCG-3', Mito probe 5'-(TET)-CTAGTCTTTGCCGCCTGCGAAGCA-(TAMRA)-3' [91].

## Competing interests

The authors declare that they have no competing interests.

## Authors' contributions

TP, CR, and JL conceived and designed the experiments, analyzed the data, and wrote the paper. TP and CR performed the experiments. All authors read and approved the final manuscript.

## Supplementary Material

Additional file 1 **Figure S1. SIV**_**MAC **_***vpx***^**+ **^**VLPs do not disrupt innate immune responses in MDDC**. (**A**) MDDCs were treated with *vpx*^+ ^or Δ*vpx *SIV_MAC-251 _VLPs or media as a control for 3 h, and then treated with the indicated compounds for 24 h. Upregulated surface expression of CD86 on MDDC was then determined by flow cytometry. (**B**) MDDCs were treated with *vpx*^+ ^or Δ*vpx *SIV_MAC-251 _VLPs for 3 h, and then treated with LPS for 24 h. The MDDC media was then collected and added to HL116 cells, which carry the luciferase gene under the control of the IFN-inducible 6-16 promoter, for 7 h. The HL116 cells were then subjected to a luciferase assay to quantify endogenous IFN-β protein levels in the MDDC media, as compared to a standard curve of known, recombinant IFN-β levels. (**C**) MDDCs were treated with *vpx*^+ ^or Δ*vpx *SIV_MAC-251 _VLPs for 3 h, and then treated with the indicated compounds for 24 h. Whole-cell lysates were prepared from MDDC and subjected to SDS-PAGE/western blot analysis. Membranes were probed with the indicated antibodies. (**D**) MDDCs were treated with *vpx*^+ ^or Δ*vpx *SIV_MAC-251 _VLPs for 3 h, and then treated with LPS for 2 h. Total RNA was extracted from MDDC and subjected to qRT-PCR analysis with the indicated Taqman-based gene expression assays. Data represent one of at least three independent experiments. Error bars represent ± SD (*n *= 3). Click here for file

Additional file 2 **Figure S2. Vpx is necessary to rescue HIV-1 from the type I IFN response in MDDC**. (A) MDDCs were treated with the indicated compounds for 24 h and then treated for 3 h with media or VSV-G-pseudotyped SIV_MAC-251 _VLPs where *vpx *is either supplied *in cis *or *in trans*, or where *vpx *is deleted entirely. 72 h after challenge with a VSV-G-pseudotyped HIV-1_NL4-3 _GFP reporter virus, the MDDC were assayed by flow cytometry for GFP expression. (**B**) MDDCs were treated with the indicated compounds for 24 h and then treated for 3 h with media, or with the indicated VSV-G-pseudotyped SIV_MAC-239 _luciferase reporter viruses, and then challenged with a VSV-G-pseudotyped HIV-1_NL4-3 _GFP reporter virus. MDDC were analyzed by flow cytometry 72 h after transduction. Data represent one of at least three independent experiments. Error bars represent ± SD (*n *= 3). Click here for file

Additional file 3 **Figure S3. Vpx does not protect SIV**_**MAC **_**or HIV-2 from the type I interferon response in MDDC**. MDDCs were treated with LPS for 24 h and then treated with media, or the indicated *vpx*^+ ^or Δ*vpx *SIV_MAC-251 _or HIV-2_ROD _VLPs for 3 h. The MDDC were then challenged with either a SIV_MAC-251 _(**A**) or HIV-2_ROD _(**B**) GFP reporter vector. MDDCs were analyzed by flow cytometry 72 h after transduction. Data represent one of at least three independent experiments. Error bars represent ± SD (*n *= 3). Click here for file

Additional file 4 **Figure S4. Vpx does not disrupt innate immune signaling**. HEK-293 cells were transfected with codon-optimzed SIV_MAC-251 _*vpx *or empty pcDNA3.1 plasmid as a control, along with a luciferase reporter plasmid for *IFNΒ1 *(**A**), NF-κB (**B**), or AP-1 (**C**), and an expression plasmid for MAVS (**A**), MyD88 (**B**), or TAB3 (**C**). Cells were harvested for luciferase assay 48 h post-transfection. Data are normalized to a *Renilla *luciferase internal control and are representative of one of at least three independent experiments. Error bars represent ± SD (*n *= 6). Click here for file

Additional file 5 **Table S1. Codon-optimized nucleic acid sequences**. Click here for file

Additional file 6 **Table S2. Oligonucleotides used for cloning in this study**. Click here for file
