# Supplementary figures and images for: Vpx rescues HIV-1 transduction of dendritic cells from the antiviral state established by type 1 interferon
Source: Retrovirology. 2011 Jun 22;8:49. doi: 10.1186/1742-4690-8-49 (PMC3130655; doi:10.1186/1742-4690-8-49)

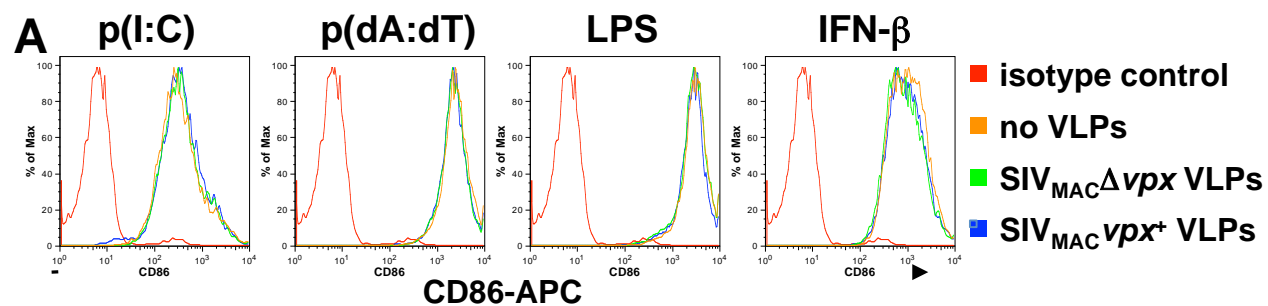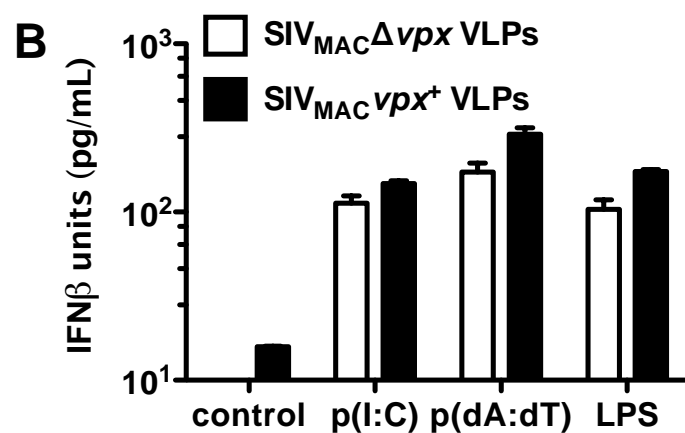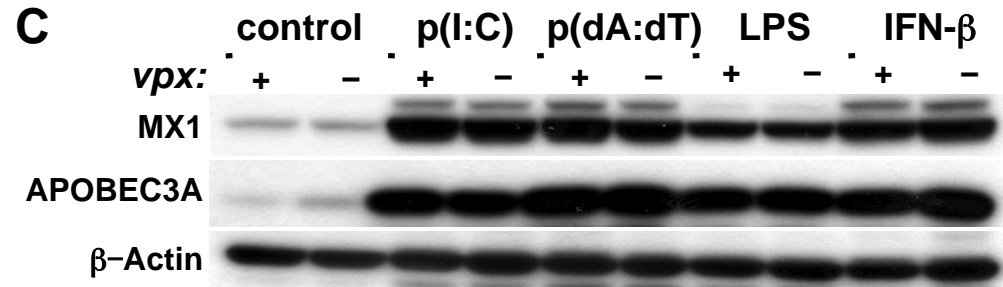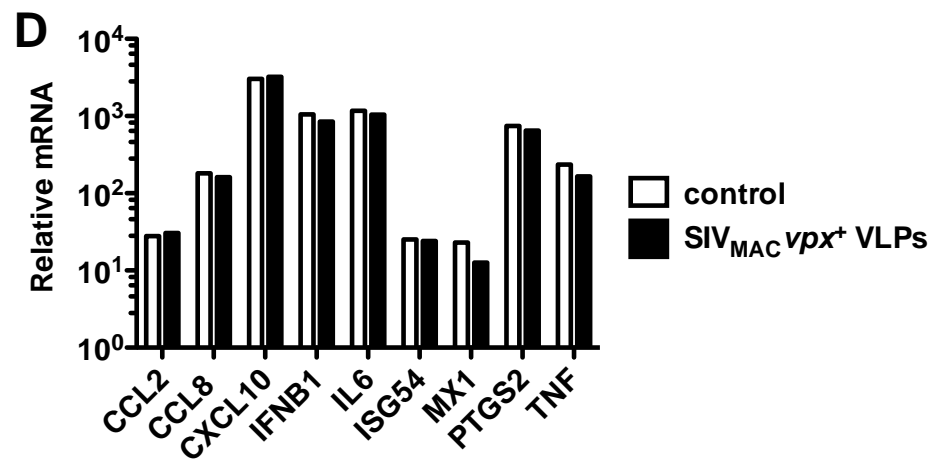

Supplement: Additional file 1 — Figure S1. SIVMAC vpx+ VLPs do not disrupt innate immune responses in MDDC. (A) MDDCs were treated with vpx+ or Δvpx SIVMAC-251 VLPs or media as a control for 3 h, and then treated with the indicated compounds for 24 h. Upregulated surface expression of CD86 on MDDC was then determined by flow cytometry. (B) MDDCs were treated with vpx+ or Δvpx SIVMAC-251 VLPs for 3 h, and then treated with LPS for 24 h. The MDDC media was then collected and added to HL116 cells, which carry the luciferase gene under the control of the IFN-inducible 6-16 promoter, for 7 h. The HL116 cells were then subjected to a luciferase assay to quantify endogenous IFN-β protein levels in the MDDC media, as compared to a standard curve of known, recombinant IFN-β levels. (C) MDDCs were treated with vpx+ or Δvpx SIVMAC-251 VLPs for 3 h, and then treated with the indicated compounds for 24 h. Whole-cell lysates were prepared from MDDC and subjected to SDS-PAGE/western blot analysis. Membranes were probed with the indicated antibodies. (D) MDDCs were treated with vpx+ or Δvpx SIVMAC-251 VLPs for 3 h, and then treated with LPS for 2 h. Total RNA was extracted from MDDC and subjected to qRT-PCR analysis with the indicated Taqman-based gene expression assays. Data represent one of at least three independent experiments. Error bars represent ± SD (n = 3). [file 1742-4690-8-49-S1.PDF]

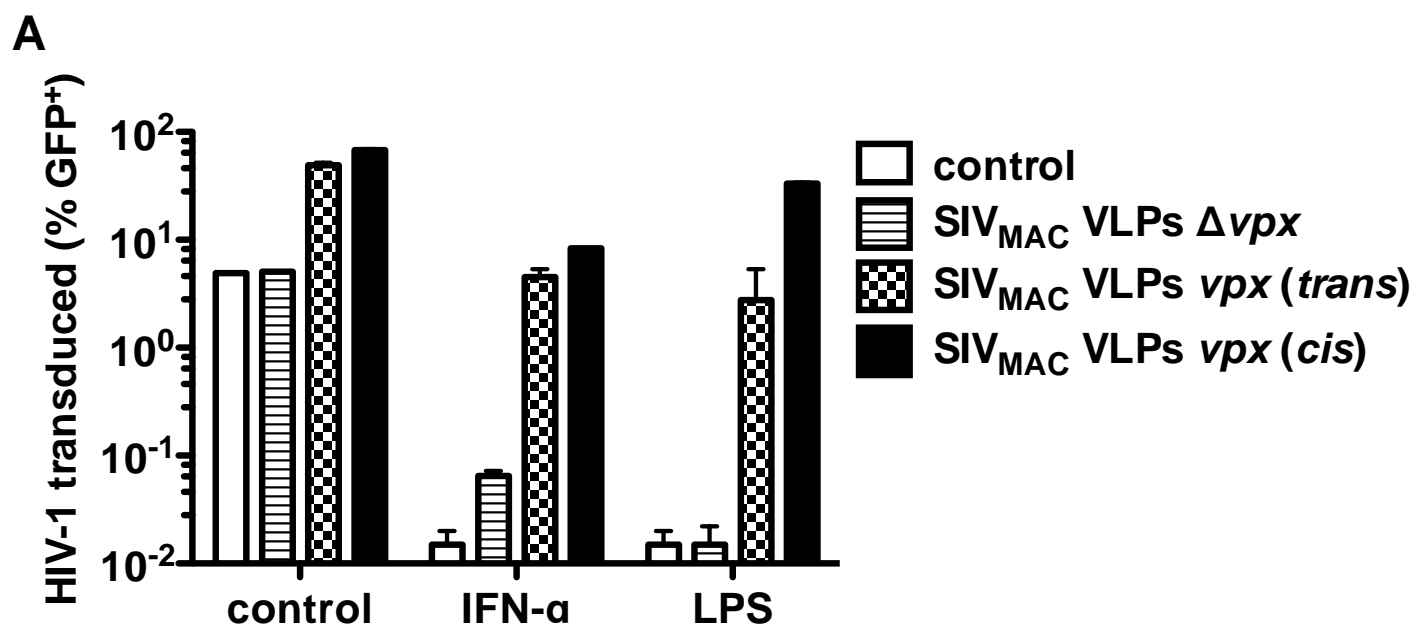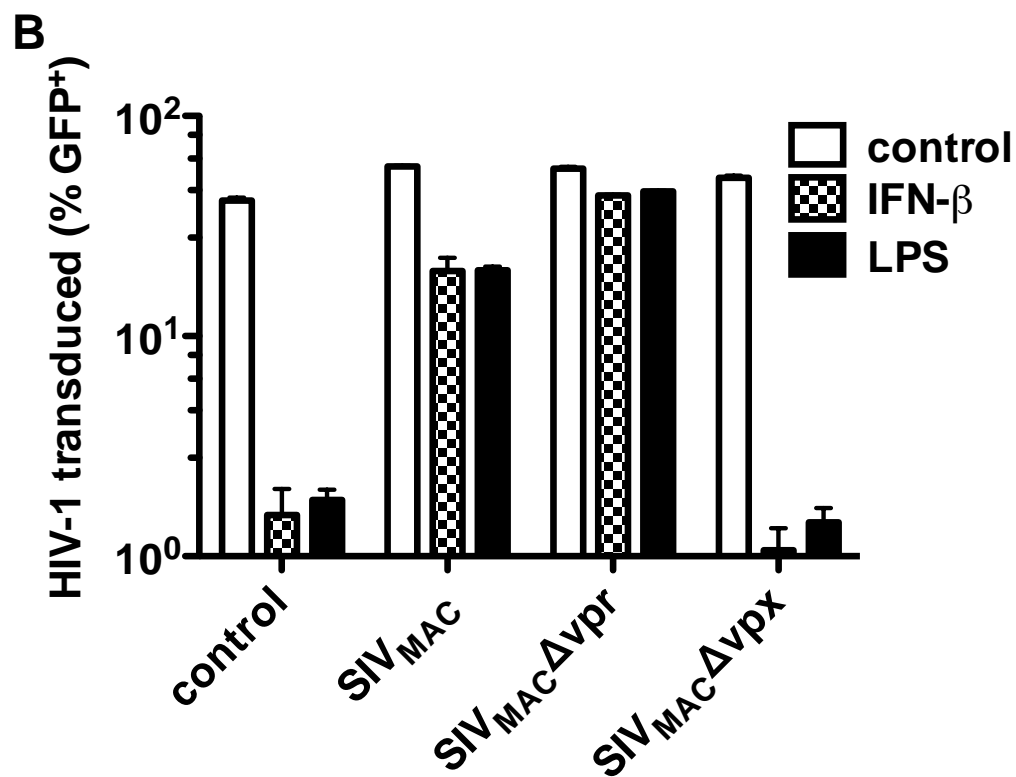

Supplement: Additional file 2 — Figure S2. Vpx is necessary to rescue HIV-1 from the type I IFN response in MDDC. (A) MDDCs were treated with the indicated compounds for 24 h and then treated for 3 h with media or VSV-G-pseudotyped SIVMAC-251 VLPs where vpx is either supplied in cis or in trans, or where vpx is deleted entirely. 72 h after challenge with a VSV-G-pseudotyped HIV-1NL4-3 GFP reporter virus, the MDDC were assayed by flow cytometry for GFP expression. (B) MDDCs were treated with the indicated compounds for 24 h and then treated for 3 h with media, or with the indicated VSV-G-pseudotyped SIVMAC-239 luciferase reporter viruses, and then challenged with a VSV-G-pseudotyped HIV-1NL4-3 GFP reporter virus. MDDC were analyzed by flow cytometry 72 h after transduction. Data represent one of at least three independent experiments. Error bars represent ± SD (n = 3). [file 1742-4690-8-49-S2.PDF]

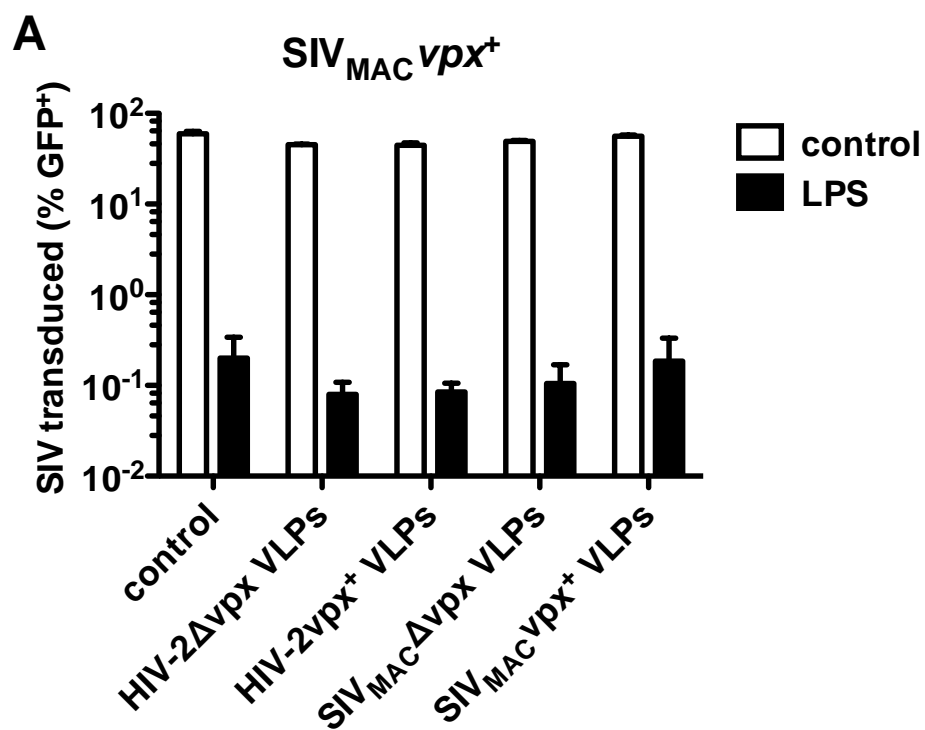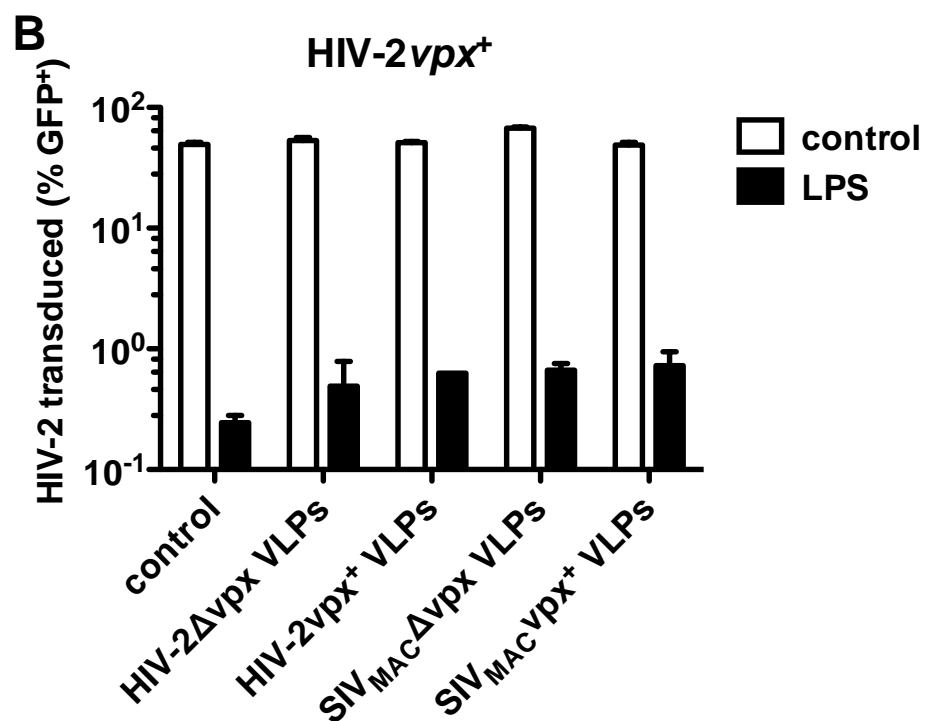

Supplement: Additional file 3 — Figure S3. Vpx does not protect SIVMAC or HIV-2 from the type I interferon response in MDDC. MDDCs were treated with LPS for 24 h and then treated with media, or the indicated vpx+ or Δvpx SIVMAC-251 or HIV-2ROD VLPs for 3 h. The MDDC were then challenged with either a SIVMAC-251 (A) or HIV-2ROD (B) GFP reporter vector. MDDCs were analyzed by flow cytometry 72 h after transduction. Data represent one of at least three independent experiments. Error bars represent ± SD (n = 3). [file 1742-4690-8-49-S3.PDF]

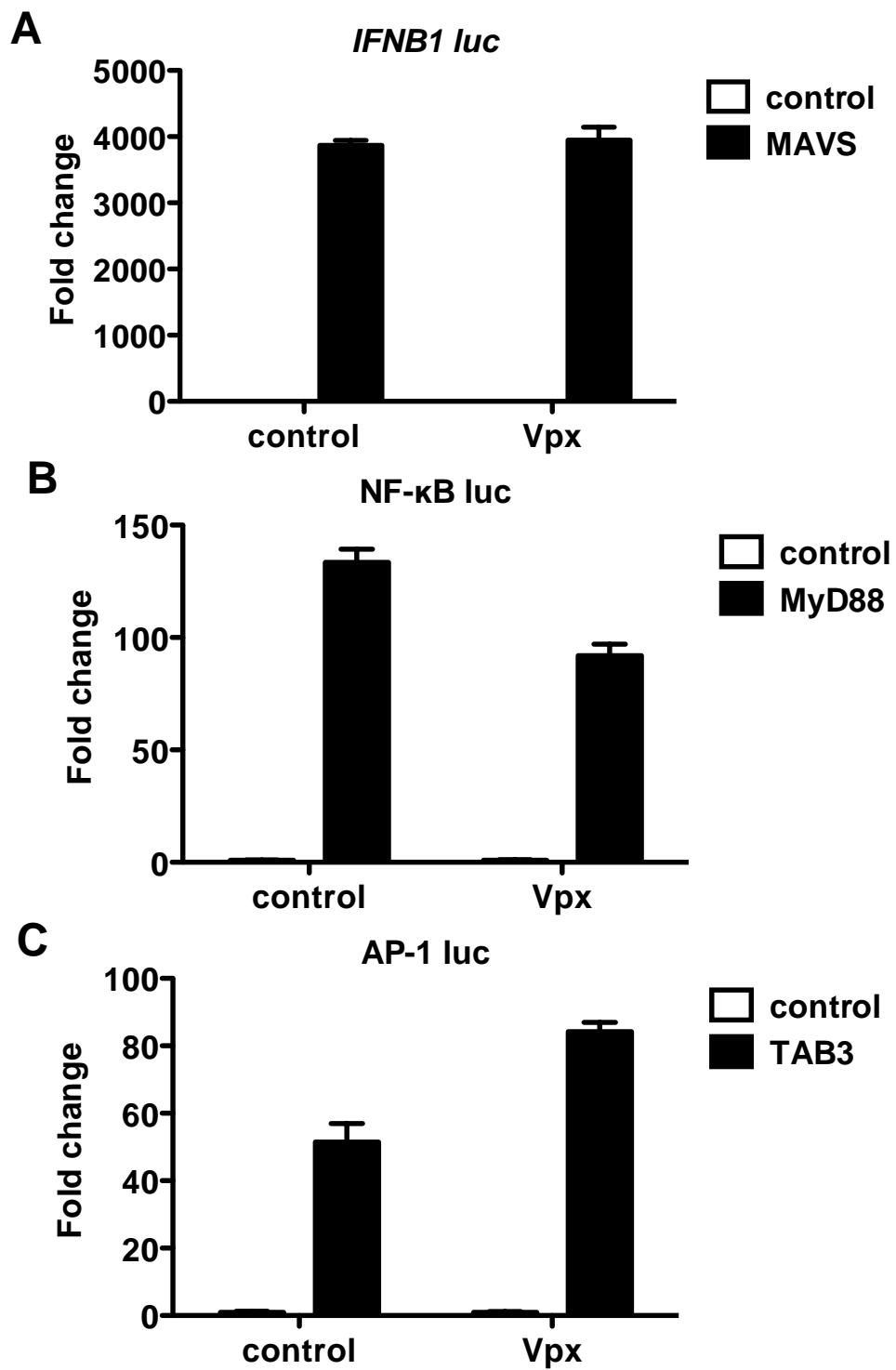

Supplement: Additional file 4 — Figure S4. Vpx does not disrupt innate immune signaling. HEK-293 cells were transfected with codon-optimzed SIVMAC-251 vpx or empty pcDNA3.1 plasmid as a control, along with a luciferase reporter plasmid for IFNΒ1 (A), NF-κB (B), or AP-1 (C), and an expression plasmid for MAVS (A), MyD88 (B), or TAB3 (C). Cells were harvested for luciferase assay 48 h post-transfection. Data are normalized to a Renilla luciferase internal control and are representative of one of at least three independent experiments. Error bars represent ± SD (n = 6). [file 1742-4690-8-49-S4.PDF]
