# Supplementary material for: Vpx rescues HIV-1 transduction of dendritic cells from the antiviral state established by type 1 interferon
Source: Retrovirology. 2011 Jun 22;8:49. doi: 10.1186/1742-4690-8-49 (PMC3130655; doi:10.1186/1742-4690-8-49)
Supplement: Additional file 5 — Table S1. Codon-optimized nucleic acid sequences. [file 1742-4690-8-49-S5.PDF]

Additional file 5

Table S1. Codon-optimized nucleic acid sequences.

**Accessory gene**	**Codon-optimized nucleic acid sequence**
$SIV_{MAC-251}$ *vpx*	atgagcgacccaagagaaagaatcccacctggaaatagcggcgaagaaactat tggagaggctttcgagtggctgaatagaaccgtggaggagataaatagagaagct gtgaaccatctgcccagagagctgatcttccaagtgtggcaaaggagctgggagt attggcacgacgagcagggcatgtcccagagctatgtgaaatatagatatctgtgtc tgatgcagaaggcactgttcatgcactgtaaaaagggctgtaggtgcctcggggaa ggacatggggccggcggatggaggcccggcccacctcctccccctcccccggc ctcgcatga
$HIV\text{-}2_{ROD}$ *vpx*	atgacagatccacgagagaccgtacccccaggcaacagtggagaagaaaccat tggcgaggcgttcgcatggctcaacaggacggtggaggccatcaacagagaag ccgtaaatcacctgcccagggaacttatctttcaggtctggcagaggagctggcggt actggcacgacgagcagggcatgtctgagagctataccaaataccgctacctttgt atcatccagaaggccgtttacatgcacgtgagaaaaggatgtacatgcttgggaag aggtcacggccctggcggctggagacctggcccaccaccccctcccccacctgg gctggtgtga
$SIV_{SMM-PBj}$ *vpx*	atgtctgatcccagggagaggattccaccaggaaattcaggggaggagaccatc ggtgaggcattcgactggttggatcgcacagtggaggaaatcaatcgggccgcag taaaccatttgccacgcgaacttatttttcaggtgtggcggcgcagttgggagtattgg tacgacgagatgggcatgtctgtgagttatacgaagtacagatacttgtgtcttatcca gaaggccatgttcatgcactgcaagaaaggctgccggtgtctcgggggagagca cggcgcgggcggctggagacctggcccaccaccccctcccccacctgggctggc ctga
$SIV_{AGM-TAN}$ *vpr*	atggcatctggcagagatccaagggaaccattgccgggatggctggagatatggg accttgaccgtgagccgtgggatgagtggctccaagatatgcttagggatctgaac gaagaggcgcgaagacactttggtatgaatatgttgatccgggtgtggaattattgc gtcgaggaagggagaaggcacaatacccccctggaacgagataggatacaaata ctacaggatagtgcagaagagcatgtttgtgcactttcgttgcggctgtaggcgacg gggcccttttccccgtatgaagagaggagaaacggccagggtggaggagcccc acctcccccacctgggctggcctga
