# Supplementary material for: Vpx rescues HIV-1 transduction of dendritic cells from the antiviral state established by type 1 interferon
Source: Retrovirology. 2011 Jun 22;8:49. doi: 10.1186/1742-4690-8-49 (PMC3130655; doi:10.1186/1742-4690-8-49)
Supplement: Additional file 6 — Table S2. Oligonucleotides used for cloning in this study. [file 1742-4690-8-49-S6.PDF]

Additional file 6

Table S2. Oligonucleotides used for cloning in this study.

**Primer name**	**Primer sequence**
coHIV-2rod Vpx NotI 3'	5'-ACCAGCGGCCGCTCACACCAGCCCAG-3'
coHIV-2rod Vpx XbaI 5'	5'-CAACTCTAGAGCCACCATGACAGATCCACGAG-3'
coSIVagm-tan Vpr NotI 3'	5'-ACCAGCGGCCGCTCAGGCCAGCCCAGGTG-3'
coSIVagm-tan Vpr XbaI 5'	5'-CAACTCTAGAGCCACCATGGCATCTGGCAGAGA-3'
coSIVmac-251 Vpx dPro NotI 3'	5'-ACCAGCGGCCGCTCAGGGCCTCCATCCGCC-3'
coSIVmac-251 Vpx F80A 3'	5'-ACAGTGCATTGCCAGTGCCTT-3'
coSIVmac-251 Vpx F80A 5'	5'-AAGGCACTGGCAATGCACTGT-3'
coSIVmac-251 Vpx K77A 3'	5'-GAACAGTGCGGCCTGCATCAG-3'
coSIVmac-251 Vpx K77A 5'	5'-CTGATGCAGGCCGCACTGTTC-3'
coSIVmac-251 Vpx Not 3'	5'-ACCAGCGGCCGCTCATGCGAGGCCGGGGGA-3'
coSIVmac-251 Vpx Q76A 3'	5'-CAGTGCCTTGGCCATCAGACA-3'
coSIVmac-251 Vpx Q76A 5'	5'-TGTCTGATGGCCAAGGCACTG-3'
coSIVmac-251 Vpx Xba 5'	5'-CAACTCTAGAGCCACCATGAGCGACCCAAGAGAAAGAA-3'
coSIVsmm-pbj Vpx NotI 3'	5'-ACCAGCGGCCGCTCAGGCCAGCCCAGGTGG-3'
coSIVsmm-pbj Vpx XbaI 5'	5'-CAACTCTAGAGCCACCATGTCTGATCCCAG-3'
